# Reversible Decarbonylation of a Metal Phosphaketene

**DOI:** 10.1002/chem.202501527

**Published:** 2025-05-24

**Authors:** Matthew J. Reveley, Joey Feld, Jose M. Goicoechea

**Affiliations:** ^1^ Department of Chemistry University of Oxford Chemistry Research Laboratory 12 Mansfield Rd. Oxford OX1 3TA UK; ^2^ Department of Chemistry Indiana University 800 East Kirkwood Ave. Bloomington IN 47405 USA

**Keywords:** cobalt, phosphaethynolate, phosphastannene, phosphorus, pyridine diimine

## Abstract

The synthesis of a metalated phosphastannene by reaction of the known cobalt(I) phosphaketene complex Co(^Dipp^PDI)(PCO) (^Dipp^PDI = 1,1′‐(pyridine‐2,6‐diyl)bis(*N*‐(2,6‐diisopropylphenyl)ethan‐1‐imine) with Sn[CH(SiMe_3_)_2_]_2_ is described. The resulting compound, Co(^Dipp^PDI)(CO){P═Sn[CH(SiMe_3_)_2_]_2_}, contains a localized P═Sn double bond, yet reacts like a phosphaketene transfer reagent, suggesting that, in solution, Co(^Dipp^PDI)(CO){P═Sn[CH(SiMe_3_)_2_]_2_} appears to exist in equilibrium with Co(^Dipp^PDI)(PCO) and Sn[CH(SiMe_3_)_2_]_2_. This is facilitated by the fact that, on decarbonylation with Sn[CH(SiMe_3_)_2_]_2_, the carbon monoxide extruded from the phosphaketene precursor Co(^Dipp^PDI)(PCO) remains in the coordination sphere of cobalt. The novel phosphaketene compounds (IDipp)Au(PCO) (IDipp = 1,3‐bis(2,6‐diisopropylphenyl)imidazol‐2‐ylidene) and (^Dipp^NacNac)Zn(PCO) (^Dipp^Nacnac = HC[C(Me)N(Dipp)]_2_; Dipp = 2,6‐*^i^* Pr_2_C_6_H_3_) can be synthesized by reaction of Co(^Dipp^PDI)(CO){P═Sn[CH(SiMe_3_)_2_]_2_} with Au(IDipp)Cl or (^Dipp^NacNac)ZnCl·LiCl(OEt_2_)_2_, respectively. In the case of the former, the released stannylene Sn[CH(SiMe_3_)_2_]_2_ ultimately inserts into the Au─PCO bond, affording the tin(IV) complex (IDipp)AuSn(PCO)[CH(SiMe_3_)_2_]_2_.

## Graphical abstract

The synthesis of a metalated phosphastannene through the base‐induced decarbonylation of a metal phosphaethynolate complex is described. This decarbonylation reaction appears to be reversible, allowing for the transfer of the phosphaethynolate moiety to other metal centers through salt‐metathesis reactions with concomitant release of a stannylene.

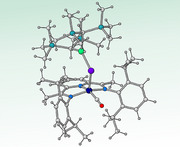

## 1 Introduction

Compounds that possess multiple bonds between the heavier (*n* >2) p‐block elements have been of fundamental interest for over fifty years. Seminal examples, which include species with Sn═Sn, Si═Si, and P═P double bonds, were first reported by Lappert,^[^ ^1^, ^2^ ^]^ West,^[^ ^3^ ^]^ and Yoshifuji,^[^ ^4^ ^]^ respectively. This pioneering work paved the way for the synthesis of a wide range of compounds with homonuclear multiple bonds.^[^ ^5^ ^]^ Unlike their lighter organic congeners (alkenes and alkynes), the weak p_π_‐p_π_ bonds in these species typically require kinetic and thermodynamic stabilization by sterically demanding substituents.^[^ ^6^, ^7^ ^]^ In recent years, renewed attention has been paid to such compounds on account of their ability to activate small molecules such as dihydrogen, carbon dioxide, and even dinitrogen.^[^ ^8^, ^9^, ^10^ ^]^

**Figure 1:**
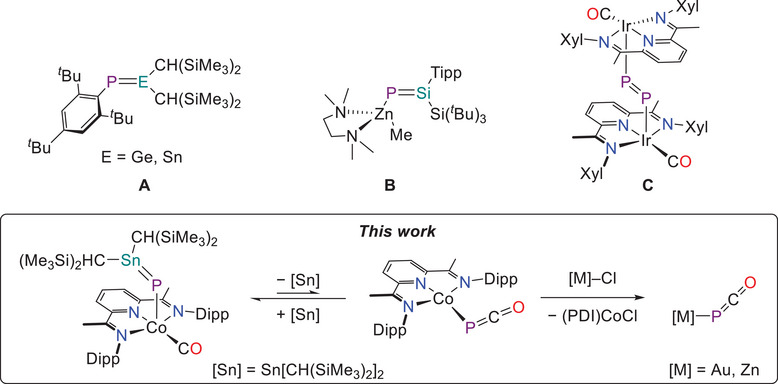
Examples of compounds with Ge═P and Sn═P double bonds derived from base‐stabilized phosphinidenes **A**),^[^ ^26^ ^]^ structurally authenticated zinc phosphasilene **B**),^[^ ^30^ ^]^ an iridium diphosphene complex formed from an iridium phosphaketene **C**).^[^ ^33^ ^]^ Tipp = 2,4,6‐*^i^* Pr_3_C_6_H_2_; Xyl = 2,6‐Me_2_C_6_H_3_.

The synthesis of compounds with *heteronuclear* multiple bonds is more challenging than their *homonuclear* counterparts and was historically achieved through dehydro‐fluorination reactions of compounds possessing a preexisting heteroatomic σ‐bond.^[^ ^11^ ^]^ In recent years, an alternate strategy using low‐valent main group nucleophiles has been proven to be particularly effective in accessing heteroatomic multiple bonds. For example, E^III^═N (E^III^ = group 13 element) bonds were readily prepared by Power and co‐workers by reaction of organic azides with group 13 carbenoids with concomitant formation of dinitrogen.^[^ ^12^, ^13^ ^]^ Base‐stabilized phosphinidenes (R─P═L; L═CO, PMe_3_) have also recently been shown to be a versatile synthon for compounds containing E═P double bonds using a similar strategy.^[^ ^14^ ^]^ Our group, and others, have reported CO or PMe_3_ displacement from these phosphinidene sources by the addition of Ga(^Dipp^NacNac) (^Dipp^NacNac = HC[C(Me)NDipp]_2_; Dipp = 2,6‐*^i^* Pr_2_C_6_H_3_) or GaCp* (Cp* = C_5_Me_5_) to afford phosphagallenes.^[^ ^15^, ^16^, ^17^, ^18^, ^19^ ^]^ This approach is also amenable for the synthesis of phosphaalumenes, using either Al(^Dipp^NacNac) or AlCp*.^[^ ^20^, ^21^ ^]^ Most recently, compounds with In═P double bonds have also been accessed using this methodology.^[^ ^22^ ^]^ The E^III^═P bonds in these compounds have been shown to react with a variety of industrially relevant small molecules, including ammonia, carbon dioxide, and alkynes.^[^ ^23^, ^24^, ^25^ ^]^ We recently extended the scope of this approach to allow access to compounds with Ge═P and Sn═P bonds (Figure 1A), which react not only as species with unsaturated heteroatomic bonds, but also as base‐stabilized phosphinidenes.^[^ ^26^ ^]^

Despite the progress made over the last decade in the synthesis of compounds with E═P bonds, the incorporation of transition metal fragments adjacent to an E═P bond remains rare. Such compounds are of interest as they may allow for cooperative reactivity by combining the unique reactivity profiles of both transition metals and unsaturated main group bonds. Metal coordination to E^IV^═P bonds (E^IV^ = Si─Sn) is viable,^[^ ^27^ ^]^ but can often lead to deleterious reactivity.^[^ ^28^ ^]^ Rarer still are E^IV^═P double bonds that are covalently bonded to a transition metal fragment, and to the best of our knowledge, only two such examples have been reported (one of which has been structurally authenticated; Figure 1B).^[^ ^29^, ^30^ ^]^ In recent years, the synthesis of [Na(dioxane)*_x_*][PCO] has enabled facile access to several d‐block phosphaketenes by salt metathesis protocols.^[^ ^31^, ^32^, ^33^ ^]^ These compounds, although harder to stabilize than their main‐group counterparts due to a propensity for metal‐centered redox events, have been shown to react as phosphinidene sources,^[^ ^34^, ^35^, ^36^, ^37^ ^]^ and readily undergo decarbonylation to afford diphosphenes (e.g., Figure 1C).^[^ ^38^, ^39^, ^40^, ^41^, ^42^ ^]^ We were interested in exploring whether the addition of suitable nucleophiles to d‐block phosphaketenes would proceed via decarbonylation to afford novel E═P double bonds incorporating a transition metal functionality. The results of our investigations are described herein.

## 2 Results and Discussion

**Scheme 1:**
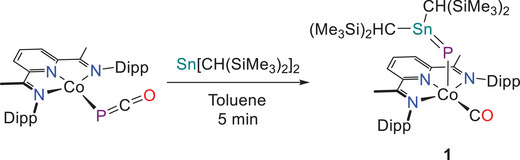
Synthesis of **1** by addition of Sn[CH(SiMe_3_)_2_]_2_ to Co(DippPDI)(PCO). Dipp = 2,6‐*^i^* Pr_2_C_6_H_3_.

The reaction of the known phosphaketene Co(^Dipp^PDI)(PCO)^[^ ^33^ ^]^ (^Dipp^PDI = 1,1′‐(pyridine‐2,6‐diyl)bis(*N*‐(2,6‐diisopropylphenyl)ethan‐1‐imine) with Sn[CH(SiMe_3_)_2_]_2_ (prepared by a modified procedure, see Supporting Information) results in a color change from purple to deep green over 5 min (Scheme 1).

**Figure 2:**
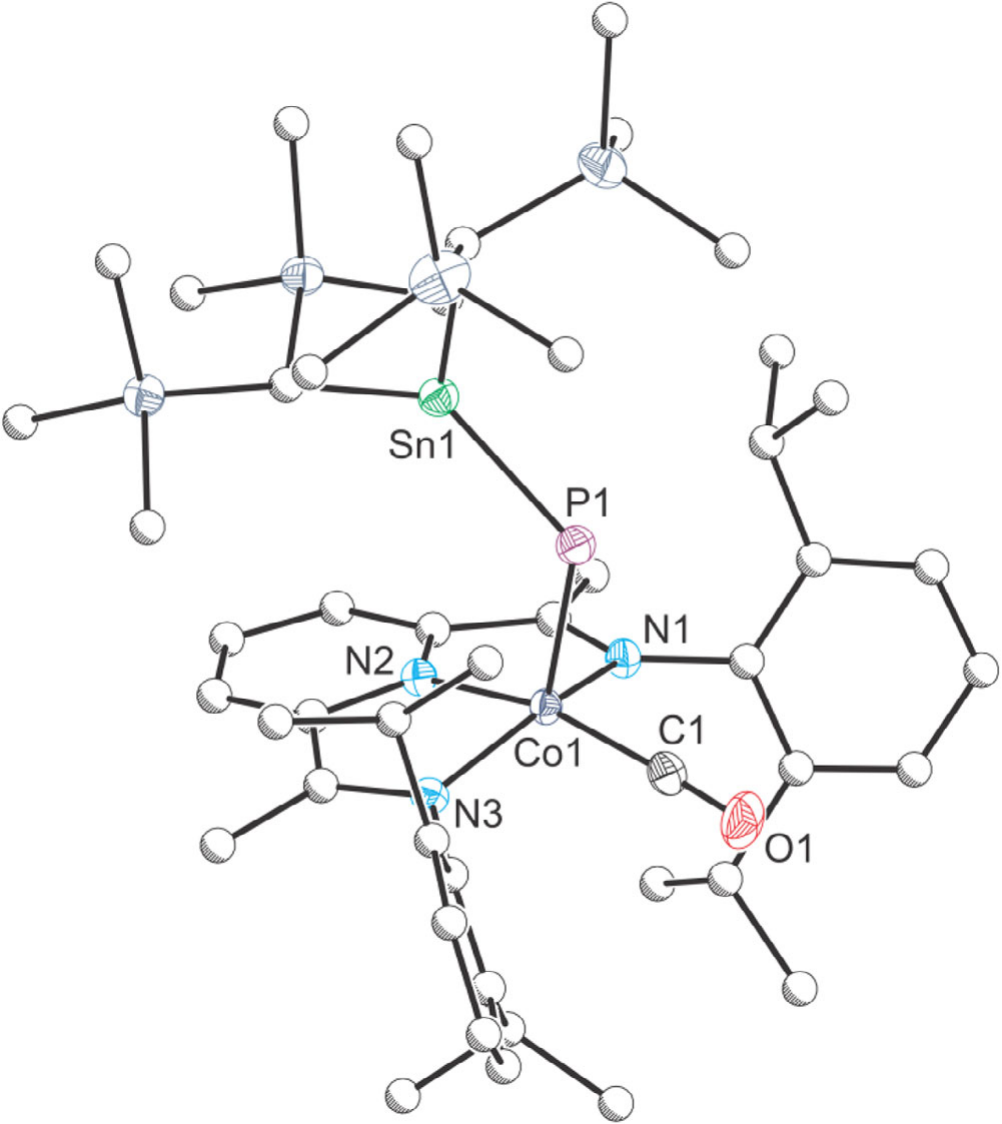
Single crystal X‐ray structure of **1**. Thermal ellipsoids set at 50% probability level; hydrogen atoms omitted for clarity. Carbon atoms (except for C1) are depicted as spheres of arbitrary radius. Selected interatomic distances [Å] and angles [°]: Co1–P1 2.3437(5), P1–Sn1 2.3335(4), Co1–N1 1.9569(14), Co1–N2 1.8192(13), Co1–N3 1.9528(13), Co1–C1 1.7691(17), C1–O1 1.138(2); Co1‐P1‐Sn1 115.680(18), Co1‐C1‐O1 177.35(16), C1‐Co1‐P1 88.42(6), C1‐Co1‐N1 97.51(7), C1‐Co1‐N2 163.78(7), C1‐Co1‐N3 95.27(7), P1‐Co1‐N1 101.94(4), P1‐Co1‐N2 107.77(4), P1‐Co1‐N3 102.70(4), N1‐Co1‐N2 80.43(6), N1‐Co1‐N3 152.46(6), N2‐Co1‐N3 80.51(6).

The formation of the cobalt phosphastannene **1** is indicated by a new resonance in the ^31^P{^1^H} nuclear magnetic resoance (NMR) spectrum at 439.3 ppm which is shifted significantly downfield from the starting material (cf. −225.8 ppm). The higher frequency of the ^31^P NMR resonance compared to other compounds with Sn═P bonds (cf. ^31^P: 202.8 ppm for **A**) is in line with the similar trend observed for the related cobalt cyaphido complex Co(^Dipp^PDI)(C≡P),^[^ ^43^ ^]^ and is presumably due to a greater paramagnetic contribution to the NMR shielding constant (σ_para_).^[^ ^44^ ^]^ The ^119^Sn NMR spectrum of **1** displays a doublet resonance at 587.2 ppm with a ^31^P─^119^Sn coupling constant of 2718 Hz, indicative of significant multiple‐bond character (cf. ^1^ *J*_Sn–P_ = 2292 Hz for **A**).^[^ ^45^ ^]^ The infrared (IR) spectrum reveals a band at 1976 cm^−1^ corresponding to a metal‐bonded carbonyl ligand, supporting transfer of carbon monoxide to the cobalt metal center.^[^ ^46^ ^]^ The identity of the product was confirmed by single‐crystal X‐ray diffraction (Figure 2).^[^ ^47^ ^]^

The Sn─P bond length is 2.3335(4) Å, which is slightly shorter than in **A**, and in line with the larger ^31^P─^119^Sn coupling constant observed for **1**.^[^ ^48^ ^]^ The complex adopts a distorted square pyramidal geometry at the cobalt center (τ_5_ = 0.189) with the Sn═P moiety occupying the axial position while the carbon monoxide fragment is retained within the plane of the PDI ligand, similarly to the iridium diphosphene **C**.^[^ ^33^ ^]^ The tin center adopts a trigonal planar geometry, supporting the formation of a Sn═P double bond (∑^°^(Sn) = 360.0°).

The ^Dipp^PDI ligand in **1** is known to display redox non‐innocence in Co(^Dipp^PDI)(L) complexes due to a low‐lying π* orbital that can be thermally populated at room temperature.^[^ ^49^ ^]^ Although formally cobalt(I) complexes, these can be described as being composed of a Co(I) center with a neutral ^Dipp^PDI ligand or, alternatively, as Co(II) centers bonded to a (^Dipp^PDI)^·−^ radical anion.^[^ ^50^ ^]^ The electronic nature of the ^Dipp^PDI ligand is dependent on the π‐accepting character of the ligand L, with good π‐acceptors favoring the neutral state of the ligand. The electronic configuration at the ^Dipp^PDI ligand is reflected in the solid‐state C─C and C─N bond lengths, and summarized by the structural parameter δ(PDI).^[^ ^51^ ^]^ For complex **1**, the δ(PDI) value is 0.079(1), which is somewhat lower than Co(^Dipp^PDI)(PCO) (δ(PDI) = 0.092(2)). The δ(PDI) values for both of these species are similar to previous examples of complexes best described as Co(II) species supported by a (^Dipp^PDI)^·−^ radical anion.^[^ ^51^ ^]^ This is surprising given that **1** also retains a strongly π‐accepting carbonyl ligand. However, this value should be interpreted with caution as, to the best of our knowledge, there are only two examples of pentacoordinate complexes with a cobalt center in the formal +1 oxidation state. For comparison, the δ(PDI) parameter for [Co(^Dipp^PDI)(*^t^* BuNC)_2_][BAr^F^_4_] (BAr^F^_4_ = tetrakis(3,5‐bis(trifluoromethyl)phenyl)borate) is 0.103(2).^[^ ^50^ ^]^

The ^1^H and ^13^C{^1^H} NMR spectra obtained for **1** agree with the obtained solid‐state geometry, including a loss of symmetry of the Dipp isopropyl groups. The NMR spectra of recrystallized samples of **1** also indicate the presence of trace amounts of free ^Dipp^PDI ligand, which is formed by decomposition of **1** at room temperature overnight (see Supporting Information), and trace amounts of Co(^Dipp^PDI)(PCO) (*vide infra*). Despite repeated attempts, the other products formed during the decomposition of **1** have not been identified. The ^31^P{^1^H} NMR spectrum of the decomposition product reveals a single resonance at −350.6 ppm with ^117/119^Sn satellites indicative of a Sn─PCO complex; however, despite multiple attempts, we were unable to isolate this compound.

**Figure 3:**
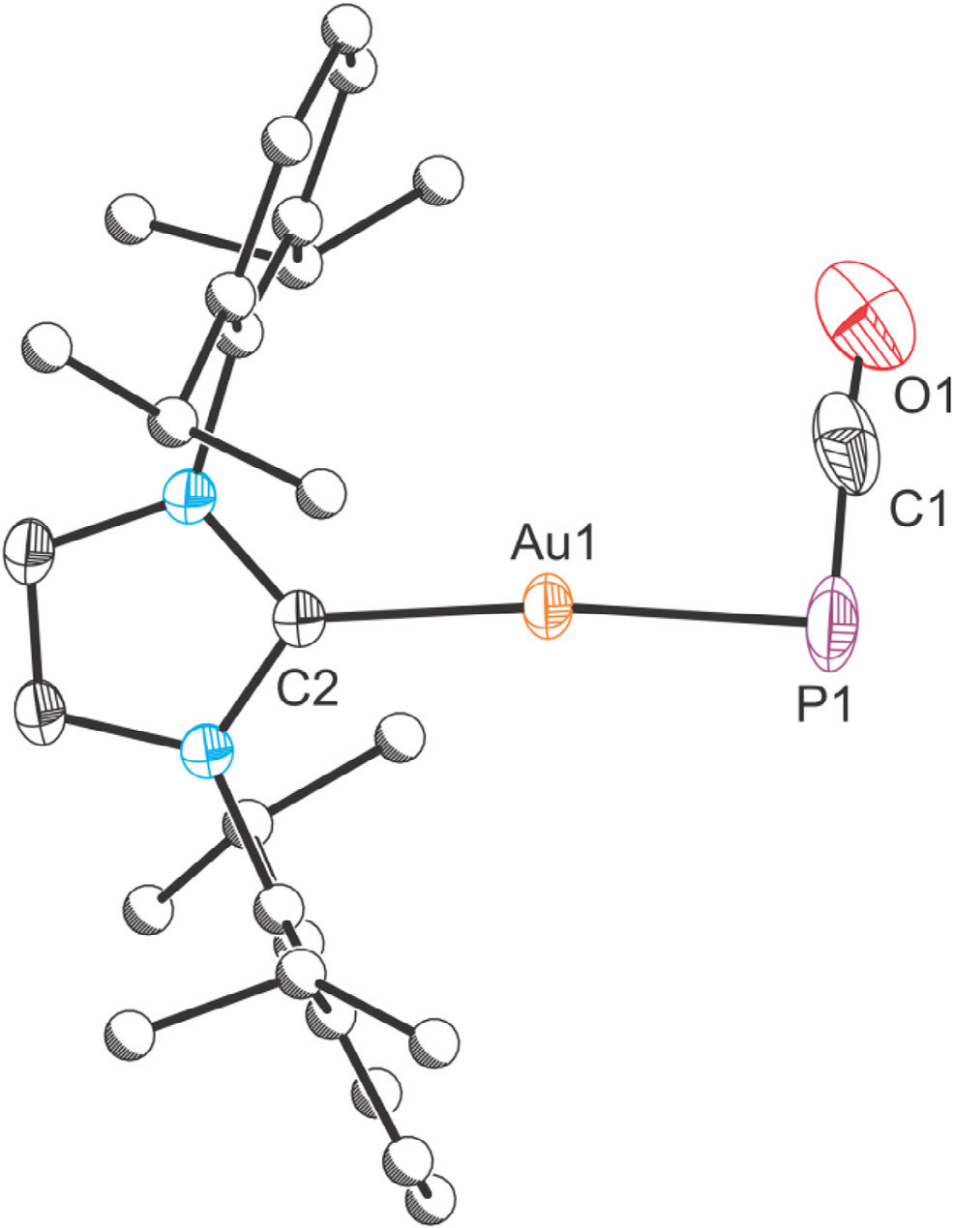
Single crystal X‐ray structure of **2**. Thermal ellipsoids set at 50% probability level; hydrogen atoms omitted for clarity. Carbon atoms of Dipp groups are depicted as spheres of arbitrary radius. Selected interatomic distances [Å] and angles [°]: Au1–P1 2.3214(11), Au1–C2 2.012(2), P1–C1 1.646(7), C1–O1 1.158(7), C2‐Au1‐P1 172.52(5), Au1‐P1‐C1 87.13(17), P1‐C1‐O1 175.9(4).

With convenient access to **1**, we aimed to investigate its reactivity. The reactivity of previous examples of Sn═P bonds is typically dominated by 1,2‐addition reactions.^[^ ^26^, ^52^, ^53^ ^]^ We therefore aimed to target novel multimetallic complexes by the addition of metal halides across the Sn═P bond.^[^ ^54^ ^]^ Addition of Au(IDipp)Cl (IDipp = 1,3‐bis(2,6‐diisopropylphenyl)imidazol‐2‐ylidene) to a C_6_D_6_ solution of **1** at room temperature did not result in the expected 1,2‐addition product. Instead, the ^31^P{^1^H} NMR spectrum reveals the formation of a new species characterized by a resonance at −361.5 ppm alongside unreacted **1**. The chemical shift of this novel compound and the presence of a doublet in its ^13^C{^1^H} NMR spectrum at 182.92 ppm are indicative of the formation of a κ‐P PCO compound (^1^ *J*_P–C_ = 102.7 Hz).^[^ ^31^ ^]^ We reasoned that the data are consistent with the formation of Au(IDipp)(PCO) (**2**). This was confirmed through an independent synthesis of the compound from the reaction of Au(IDipp)Cl with [Na(diox)_1.97_][PCO]. Compound **2** was characterized by X‐ray diffraction (Figure 3). The bond metric data for this compound are in keeping with other Au(CAAC)(PCO) compounds (where CAAC = cyclic (alkyl)(amino)(carbene)). The complex exhibits a nearly linear coordination environment at the gold(I) center (172.52(5)°), with a bent Au─PCO interaction (Au1‐P1‐C1: 87.13(17)°) as observed for other phosphaketenes.

**Scheme 2:**
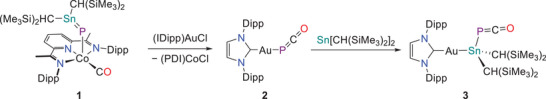
The reaction of **1** with (IDipp)AuCl yields the gold phosphaketene **2,** which is converted to the tin phosphaketene **3** by insertion into the Au─P bond.

Monitoring the reaction of Au(IDipp)Cl and **1** over several hours by ^31^P{^1^H} and ^1^H NMR spectroscopy reveals the slow conversion of **1** to **2**, and the concomitant formation of (^Dipp^PDI)CoCl. After 1 h, a third species was also observed in the ^31^P{^1^H} NMR spectrum, characterized by a resonance at −358.6 ppm. The low frequency chemical shift and observable tin satellites (^1^ *J*_Sn–P_ = 606 Hz) imply that this is a tin phosphaketene species,^[^ ^55^ ^]^ which is generated by oxidative‐addition of **2** at the tin(II) center of Sn[CH(SiMe_3_)_3_]_2_ (Scheme 2).

**Figure 4:**
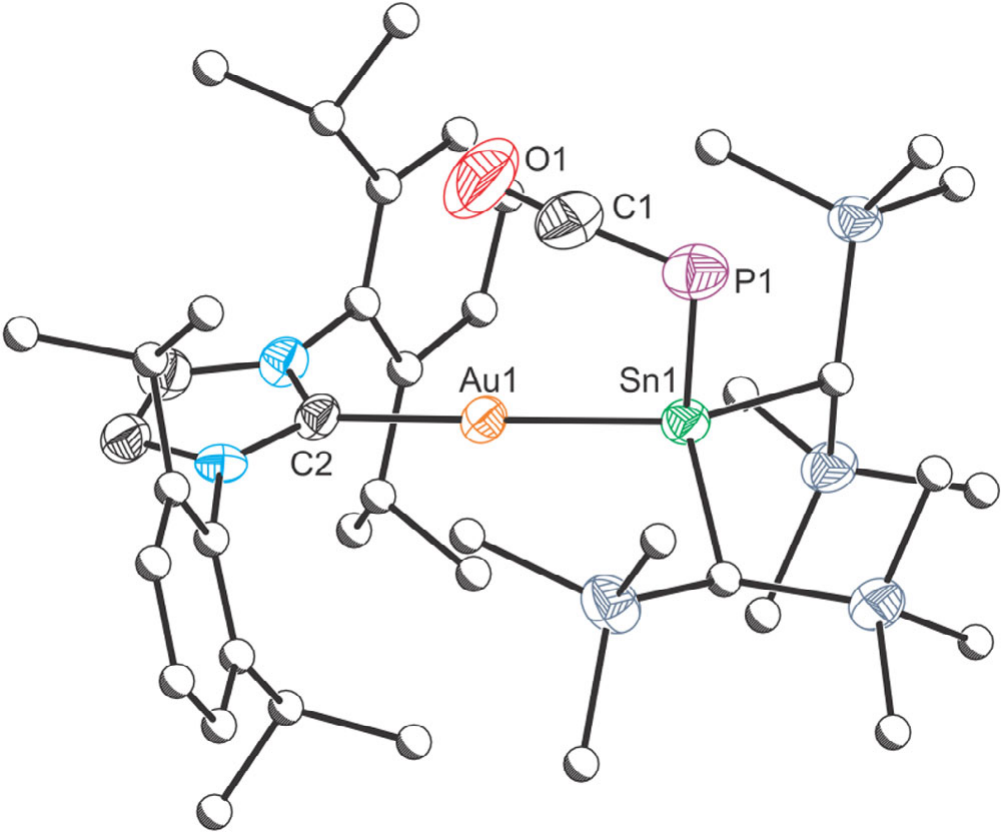
Single crystal X‐ray structure of **3**. Thermal ellipsoids set at 50% probability level; positional disorder and hydrogen atoms omitted for clarity. Carbon atoms of Dipp groups and methyl groups on SiMe_3_ are depicted as spheres of arbitrary radius. Selected interatomic distances [Å] and angles [°]: Sn1–Au1 2.5777(6), Sn1–P1 2.615(2), P1–C1 1.637(10), C1–O1 1.155(12), C2‐Au1‐Sn1 177.20(16), Sn1‐P1‐C1 89.8(3), P1‐C1‐O1 176.3(9).

To investigate the mechanism of this process, we conducted several control experiments (see Supporting Information). The reaction of (^Dipp^PDI)Co(PCO) with Au(IDipp)Cl yields a rapid transfer of the PCO moiety to form **2** alongside (^Dipp^PDI)CoCl, consistent with transmetallation reactions which have previously been reported for phosphaketenes.^[^ ^33^, ^56^ ^]^ Furthermore, the reaction of a compositionally pure sample of **2** with Sn[CH(SiMe_3_)_2_]_2_ generates **3**, in line with previous reports of stannylene insertion reactions into Au─X bonds.^[^ ^57^, ^58^ ^]^ In addition to the aforementioned ^31^P{^1^H} NMR resonance at −358.6 ppm, this compound exhibits a resonance at 183.38 ppm in its ^13^C{^1^H} NMR spectrum with a ^1^ *J*_C–P_ coupling constant of 95 Hz. This is in line with other previously reported κ‐P phosphaethynolate compounds. A doublet resonance was observed in the ^119^Sn{^1^H} NMR spectrum at 261.2 ppm. The IR spectrum of **3** reveals a band at 1836 cm^−1^ corresponding to the phosphaketene moiety. The structure of **3** was confirmed by single‐crystal X‐ray diffraction (Figure 4). As expected, it reveals a tetrahedral tin(IV) center bonded to the Au(IDipp) and phosphaketene moieties in addition to the two CH(SiMe_3_) groups. The Sn─Au and Sn─P bond distances and 2.5777(6) and 2.615(2) Å, respectively, are in line with single bonds (∑_cov_(Sn─Au) = 2.64 Å; ∑_cov_(Sn─P) = 2.51 Å).^[^ ^59^ ^]^ The coordination environment at the gold(I) center is linear (177.20(16)°). As with other phosphaketene compounds, the Sn─PCO interaction is bent and very close to 90° (89.8(3)°).

Taken together, we propose that the retention of the CO moiety by the cobalt center in **1** allows it to act as a base‐stabilized phosphinidene. Our observations of trace amounts of (^Dipp^PDI)Co(PCO) in recrystallized samples of **1** support this hypothesis, implying that the phosphaketene exists in an equilibrium which heavily favors the phosphastannene. Notably, our attempts to exchange the CO moiety in **1** by the addition of *^t^* BuNC or IMe_4_ (IMe_4_ = C[N(CH_3_)C(CH_3_)]_2_)) resulted in rapid decomposition to intractable mixtures. The addition of Au(IDipp)Cl to **1** perturbs the equilibrium toward the cobalt phosphaketene by PCO^−^ transfer to give **2**. The regenerated stannylene is then able to react with **2** to give **3** by insertion into the Au─P bond. The reformation of the stannylene in this process is consistent with our previous report of phosphinidene generation by dissociation of a Sn═P double bond to generate Sn[CH(SiMe_3_)_2_]_2_.^[^ ^26^ ^]^

**Figure 5:**
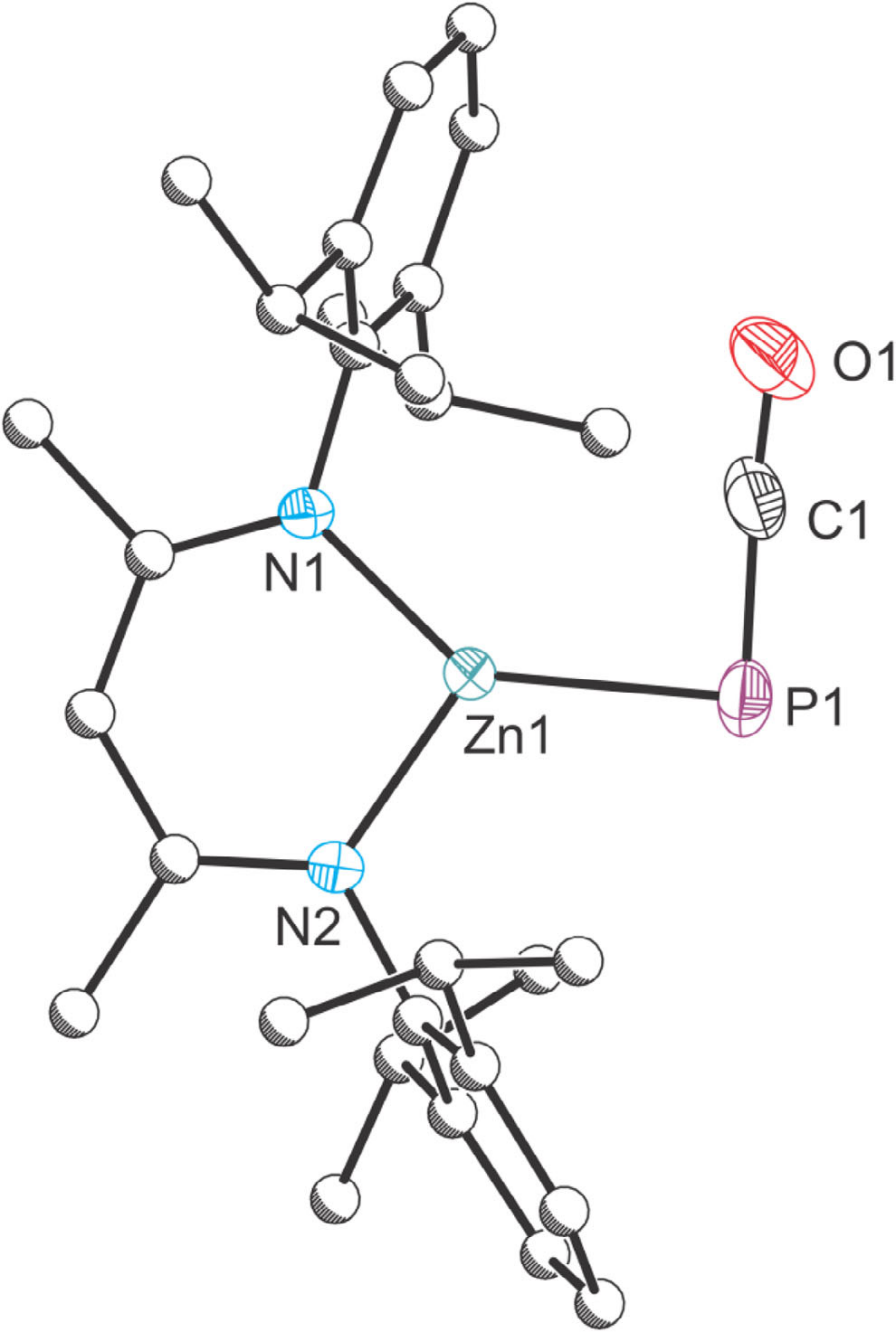
Single crystal X‐ray structure of **4**. Thermal ellipsoids set at 50% probability level; positional disorder of phosphaethynolate moiety and hydrogen atoms omitted for clarity. Carbon atoms (except for C1) are depicted as spheres of arbitrary radius. Selected interatomic distances [Å] and angles [°]: Zn1–P1 2.2804(4), Zn1–N1 1.9187(9), Zn1–N2 1.9318(9), P1–C1 1.6448(18), C1–O1 1.158(2), P1‐Zn1‐N1 140.86(3), P1‐Zn1‐N2 119.17(3), N1‐Zn1‐N2 99.95(4), Zn1‐P1‐C1 88.13(6), P1‐C1‐O1 175.68(16).

We also explored the effects of steric protection at the metal center on this reaction. The reaction of **1** with (^Dipp^NacNac)ZnCl·LiCl(OEt_2_)_2_ yielded a new species by ^31^P NMR spectroscopy characterized by a resonance at −403.1 ppm which was confirmed as (^Dipp^NacNac)Zn(PCO) (**4**) by X‐ray diffraction (Figure 5). Species **4** is the first phosphaethynolate complex of zinc. It features a planar three coordinate zinc(II) center (∑° = 360°) and a Zn─P distance, 2.2804(4) Å, consistent with a single bond (sum of single bond covalent radii = 2.29 Å).^[^ ^59^ ^]^ As with all other metallo‐phosphaketene complexes, the interaction between the zinc center and the phosphaethynolate ligand is bent (88.13(6)°). The P─C─O bond angle (175.68(16)°) and P─C and C─O bond distances of the phosphaethynolate moiety (1.6448(18) and 1.158(2) Å, respectively) are consistent with those of other phosphaketenes including, for example, compound **2** (P‐C‐O: 175.9(4)°; P─C: 1.646(7) Å; C─O: 1.158(7) Å). Compound **4** can be synthesized independently from [Na(dioxane)_x_][PCO] and (^Dipp^NacNac)ZnCl·LiCl(OEt_2_)_2_.

In line with the greater steric demand of the ^Dipp^NacNac ligand, the PCO^−^ transfer reaction with **1** occurs more slowly than for **2**, leading to some decomposition of **1** even when the zinc species is present in excess. Nevertheless, the reaction proceeds via (^Dipp^PDI)Co(PCO) (see Supporting Information), and no insertion reaction of Sn[CH(SiMe_3_)_2_]_2_ into the Zn─P bond was observed, presumably on account of the increased steric protection around the Zn─P bond.

## 3 Conclusion

In conclusion, we have prepared the first example of a compound containing a Sn═P double bond, which is bonded to a transition metal moiety at the phosphorus atom. The reactivity of the phosphastannene with metal halides is highly influenced by the cobalt center, acting as a base‐stabilized phosphinidene to enable PCO transfer. Three novel metal phosphaketenes were spectroscopically observed whilst exploring reactions of ligated metal halides with **1**, all three of which were independently synthesized using alternative strategies. Further investigations into the synthesis of other metal‐bonded E═P bonds are currently ongoing.

## Supporting Information

The authors have cited additional references within the Supporting Information.^[^ ^60^, ^61^, ^62^, ^63^, ^64^, ^65^, ^66^, ^67^, ^68^ ^]^

## Conflict of Interests

The authors declare no conflict of interest.

## Supporting information



Supporting Information

## Data Availability

The data that support the findings of this study are available in the supplementary material of this article.
