# Supplementary material for: Reversible Decarbonylation of a Metal Phosphaketene
Source: Chemistry. 2025 May 24;31(36):e202501527. doi: 10.1002/chem.202501527 (PMC12202850; doi:10.1002/chem.202501527)
Supplement: Supplementary file 1 — Supporting Information [file CHEM-31-e202501527-s001.docx]

*Supporting Information*

**Table of Contents**

[1. Experimental Section 2](#_Toc196222406)

[1.1. General synthetic methods 2](#_Toc196222407)

[1.1.1. Synthesis of [(Me_3_Si)_2_CH]_2_Sn=PCo(CO)(^Dipp^PDI) (**1**) 3](#_Toc196222408)

[1.1.2. Synthesis of (IDipp)Au(PCO) (**2**) 8](#_Toc196222409)

[1.1.3. Synthesis of [(Me_3_Si)_2_CH]_2_Sn(PCO)Au(IDipp) (**3**) 11](#_Toc196222410)

[1.1.4. Synthesis of (^Dipp^NacNac)Zn(PCO) (**4**) 15](#_Toc196222411)

[1.2.1. Alternative synthesis of Sn[CH(SiMe_3_)_2_]_2_ 18](#_Toc196222412)

[1.2.2. Decomposition of **1** above −35 °C 19](#_Toc196222413)

[1.2.3. Reaction of **1** with (IDipp)AuCl 22](#_Toc196222414)

[1.2.4. Reaction of **1** with (^Dipp^NacNac)ZnCl·LiCl(OEt_2_)_2_ 24](#_Toc196222415)

[1.2.5. Reaction of (^Dipp^PDI)Co(PCO) with (IDipp)AuCl 26](#_Toc196222416)

[1.2.6. Reaction of (^Dipp^PDI)Co(PCO) with (^Dipp^NacNac)ZnCl·LiCl(OEt_2_)_2_ 27](#_Toc196222417)

[2. Single crystal X-ray diffraction data 29](#_Toc196222418)

[3. Calculation of δ(PDI) parameters 31](#_Toc196222419)

[4. References 32](#_Toc196222420)

# 1. Experimental Section

## 1.1. General synthetic methods

All reactions and product manipulations were carried out using standard Schlenk-line techniques under an inert atmosphere of argon, or in a dinitrogen filled glovebox (MBraun UNIlab glovebox maintained at < 0.1 ppm H_2_O and < 0.1 ppm O_2_). (^Dipp^PDI)CoCl,^[60]^ (^Dipp^PDI)Co(PCO),^[33]^ (IDipp)AuCl,^[61]^ [Na(dioxane)_x_][PCO],^[62]^ (^Dipp^NacNac)ZnCl·LiCl(OEt_2_)_2_,^[63]^ and Mg(Cl)(OEt_2_)[CH(SiMe_3_)_2_]^[64]^ were prepared according to previously reported procedures. SnCl_2_ (Alfa Aesar, anhydrous 98%,) was dried *in vacuo*, stored in a glovebox, and used without further purification. Toluene (Sigma Aldrich, HPLC grade), hexane (Sigma Aldrich, HPLC grade), pentane (Sigma Aldrich, HPLC grade), Et_2_O (Sigma Aldrich, HPLC grade), and DCM (Sigma Aldrich, HPLC grade) were purified using an MBraun SPS-800 solvent system. THF (Sigma Aldrich, HPLC grade) was distilled over sodium/benzophenone. Hexamethyldisiloxane (Sigma Aldrich, ≥98.5%) and C_6_D_6_ (Aldrich, 99.5%) were dried over CaH_2_ and degassed. All dry solvents were stored under argon in gas-tight ampoules. All solvents were stored over activated 3Å molecular sieves. Samples were sonicated using a VWR ultrasonic cleaner (45 KHz).

**Additional characterization techniques:**

NMR spectra were acquired on a Bruker AVIII 400 MHz NMR spectrometer (^1^H 400 MHz, ^31^P 162 MHz), Bruker AVIII HD 500 MHz NMR spectrometer (^13^C 126 MHz, ^119^Sn 186 MHz), or Bruker Avance NEO 600 MHz NMR spectrometer with a broadband helium cryoprobe (^13^C 151 MHz). ^1^H and ^13^C NMR spectra were referenced to the most downfield solvent resonance (^1^H NMR C_6_D_6_: δ = 7.16 ppm; ^13^C NMR C_6_D_6_: δ = 128.06 ppm). ^31^P and ^119^Sn NMR spectra were externally referenced to an 85% solution of H_3_PO_4_ in H_2_O and SnMe_4_, respectively. IR spectra were acquired on a Shimadzu IR-Spirit spectrometer using a single reflection ATR accessory by drop-casting from toluene or benzene solutions under a dinitrogen atmosphere. UV/Vis measurements were recorded using a Jasco V-770 UV-Visible/NIR spectrophotometer at room temperature. Elemental analyses were carried out by London Metropolitan University (London, U.K.). Samples (approx. 10 mg) were submitted in flame-sealed glass tubes.

## 1.1.1. Synthesis of [(Me_3_Si)_2_CH]_2_Sn=PCo(CO)(^Dipp^PDI) (1)

(^Dipp^PDI)CoCl (30 mg, 0.052 mmol) and [Na(diox)_1.9_][PCO] (15 mg, 0.060 mmol) were combined in THF (3 mL) at −78 °C and the solution slowly warmed to room temperature over 10 minutes. The volatiles were immediately removed *in vacuo*. Sn[CH(SiMe_3_)_2_]_2_ (22.7 mg, 0.052 mmol) was added to the flask inside a glovebox followed by toluene (4 mL) with stirring, causing the solution to change colour from purple to deep green over 5 minutes. The stirring was continued for 5 minutes before the volatiles were removed again *in vacuo*. The residue was extracted with pentane (10 mL) and filtered to give a green solution, which was concentrated until incipient crystal formation and cooled to −35 °C for three days. The crystals were isolated by decantation and washed with cold pentane (15.6 mg, 0.015 mmol, 28.9% yield). Crystals suitable for X-ray diffraction were grown from a saturated pentane/hexamethyldisiloxane solution at −35 °C. As the complete decomposition of **1** occurs overnight at room temperature (Figure S21−22), elemental analysis was not performed.

**^1^H NMR (400 MHz, C_6_D_6_):** δ (ppm) 7.38 (d, ^3^*J*_H–H_ = 7.7 Hz, 2H; pyridine *meta*-ArC*H*), 7.20 (m, 2H; Dipp *meta*-ArC*H*), 7.12 (m, 2H; Dipp *para*-ArC*H*), 7.02 (m, 2H; Dipp *meta*-ArC*H*), 3.66 (dhept, ^TS^*J*_P–H_ = 2.9 Hz, ^3^*J*_H–H_ = 6.8 Hz, 2H; Dipp C*H*(CH_3_)_2_), 2.27 (m, 2H; Dipp C*H*(CH_3_)_2_), 1.87 (s, 6H; N=CC*H*_3_), 1.65 (d, ^3^*J*_H–H_ = 6.5 Hz, 6H; Dipp CH(C*H*_3_)_2_), 1.24 (d, ^3^*J*_H–H_ = 6.8 Hz, 6H; Dipp CH(C*H*_3_)_2_), 1.05 (d, ^3^*J*_H–H_ = 6.8 Hz, 6H; Dipp CH(C*H*_3_)_2_), 0.77 (d, ^3^*J*_H–H_ = 6.8 Hz, 6H; Dipp CH(C*H*_3_)_2_), 0.40 (s, 18H; SiC*H*_3_), 0.16 (s, 18H; SiC*H*_3_), −0.58 (s, 2H; SnC*H*). N.B. Peaks corresponding to pyridine *para*-ArC*H* were observed in 2D spectra but were not assigned in 1D spectra due to overlap with the residual solvent peak.

**^13^C{^1^H} NMR (126 MHz, C_6_D_6_):** δ (ppm) 157.34 (Imine N=*C*CH_3­_), 149.23 (Dipp *ipso*-Ar*C*), 148.27 (pyridine *ortho*-Ar*C*), 141.34 (Dipp *ortho*-Ar*C*), 141.16 (Dipp *ortho*-Ar*C*), (127.25 (Dipp *para*-Ar*C*), 125.27 (Dipp *meta*-Ar*C*), 123.56 (Dipp *meta*-Ar*C*), 122.41 (pyridine *meta*-Ar*C*), 119.05 (pyridine *para*-Ar*C*), 38.69 (d, ^2^*J*_P–C_ = 26.3 Hz; Sn*C*H), 28.14 (Dipp *C*H(CH_3_)_2_), 27.04 (d, ^6^*J*_P–C­_ = 9.5 Hz; Dipp CH(*C*H_3_)_2_), 24.99 (Dipp CH(*C*H_3_)_2_), 24.63 (d, ^6^*J*_P–C­_ = 7.7 Hz; Dipp CH(*C*H_3_)_2_), 24.35 (Dipp CH(*C*H_3_)_2_), 17.51 (N=C*C*H­_3_), 5.15 (s(br), Si*C*H_3_), 4.96 (Si*C*H­_3_).

**^31^P{^1^H} NMR (162 MHz, C_6_D_6_):** δ (ppm) 439.3 (s, *P*=Sn).

**^119^Sn NMR (186 MHz, C_6_D_6_):** δ (ppm) 587.2 (d, ^1^*J*_Sn–P_ = 2718 Hz).

**IR (solid, cm^−1^):** *ṽ* 1976 (C≡O).

**UV-vis:** λ_max_ (nm) 362, 473, 613, 773.

**Figure S1.** ^1^H NMR spectrum (400 MHz, 293 K) of **1** in C_6_D_6_. Minor resonances corresponding to free ^Dipp^PDI ligand (decomposition product) and (^Dipp^PDI)Co(PCO) are marked in grey.

**Figure S2.** ^13^C{^1^H} NMR spectrum (126 MHz, 293 K) of **1** in C_6_D_6_. Minor resonances corresponding to free ^Dipp^PDI ligand (decomposition product) and (^Dipp^PDI)Co(PCO) are marked in grey.

**Figure S3.** ^31^P{^1^H} NMR spectrum (162 MHz, 293 K) of **1** in C_6_D_6_.

**Figure S4.** ^119^Sn{^1^H} NMR spectrum (186 MHz, 293 K) of **1** in C_6_D_6_.

**Figure S5.** ATR-FTIR spectrum of **1**.

**Figure S6.** UV-visible absorption spectrum of **1** in hexane (0.096 mg/mL).

## 1.1.2. Synthesis of (IDipp)Au(PCO) (2)

(IDipp)AuCl (50 mg, 0.080 mmol) and [Na(diox)_1.97_][PCO] (45 mg, 0.176 mmol) were

stirred in toluene for 1 hour. The suspension was filtered and the solvent was removed under vacuum. The resultant powder was washed with hexane and dried (22 mg, 0.034 mmol, 42.7% yield). Crystals suitable for X-ray diffraction were grown by layering a concentrated DCM solution with pentane at −35 °C. Anal. calculated for C_28_H_36_Au_1_N_2_O_1_P_1_: C, 52.18; H, 5.63; N, 4.35. Found: C, 52.99; H, 5.40; N, 4.13. Note: **2** begins to decompose after two days in C_6_D_6_ at room temperature.

**^1^H NMR (400 MHz, C_6_D_6_):** δ (ppm) 7.19 (t, ^3^*J*_H–H_ = 7.8 Hz, 2H; *para*-ArC*H*), 7.05 (d, ^3^*J*_H–H_ = 7.8 Hz, 4H; *meta*-ArC*H*), 6.27 (s, 2H; {(NC*H*)_2_}), 2.57 (h, ^3^*J*_H–H_ = 6.9 Hz, 4H; Dipp C*H*(CH_3_)_2_), 1.43 (d, ^3^*J*_H–H_ = 6.9 Hz, 12H; Dipp CH(C*H*_3_)_2_), 1.06 (d, ^3^*J*_H–H_ = 6.9 Hz, 12H; Dipp CH(C*H*_3_)_2_).

**^13^C{^1^H} NMR (151 MHz, C_6_D_6_):** δ (ppm) 194.21 (d, ^2^*J*_P–C_ = 39.8 Hz; carbene *C*), 18.92 (d, ^1^*J*_P–C_ = 102.7 Hz, *P*CO), 145.77 (Dipp *ortho*-Ar*C*), 134.35 (Dipp *ipso*-Ar*C*), 130.98 (Dipp *para*-Ar*C*), 124.41 (Dipp *meta*-Ar*C*), 122.50 ({(N*C*H)_2_}), 29.05 (Dipp *C*H(CH_3_)_2_), 24.65 (Dipp CH(*C*H_3_)_2_), 23.99 (Dipp CH(*C*H_3_)_2_).

**^31^P{^1^H} NMR (162 MHz, C_6_D_6_):** δ (ppm) –361.6 (s, *P*CO).

**IR (solid, cm^−1^):** *ṽ* 1867 (PCO).

**Figure S7.** ^1^H NMR spectrum (400 MHz, 293 K) of **2** in C_6_D_6_.

**Figure S8.** ^13^C{^1^H} NMR spectrum (151 MHz, 293 K) of **2** in C_6_D_6_. Inset: P*C*O resonance.

**Figure S9.** ^31^P{^1^H} NMR spectrum (162 MHz, 293 K) of **2** in C_6_D_6_.

**Figure S10.** ATR-FTIR spectrum of **2**.

## 1.1.3. Synthesis of [(Me_3_Si)_2_CH]_2_Sn(PCO)Au(IDipp) (3)

(IDipp)Au(PCO) was prepared *in situ* by sonicating a suspension of (IDipp)AuCl (50 mg, 0.080 mmol) and [Na(diox)_3.15_][PCO] (30 mg, 0.083 mmol) in toluene (5 mL) for 20 minutes to ensure complete conversion. The suspension was stirred for a further 40 minutes before the volatiles were removed *in vacuo*. Inside a glovebox, Sn[CH(SiMe_3_)_2_]_2_ (35.2 mg, 0.080 mmol) was added to the residue along with toluene (5 mL), causing the red solution to quickly fade to yellow. After stirring for 1 h, the volatiles were removed *in vacuo* and the residue extracted with pentane (6 mL). Concentration of the solution until incipient crystal formation, followed by storage at −35 °C for 3 days yielded very pale yellow crystals which were washed with cold pentane (29.3 mg, 0.027 mmol, 33.9% yield). Crystals suitable for X-ray diffraction were from a concentrated pentane solution at −35 °C. Anal. calculated for C_42_H_74_Au_1_N_2_O_1_P_1_Si_4_Sn_1_: C, 46.62; H, 6.89; N, 2.59. Found: C, 45.78; H, 7.06; N, 2.29.

**^1^H NMR (400 MHz, C_6_D_6_):** δ (ppm) 7.24 (m, 2H; Dipp *para*-C*H*), 7.12 (d, ^3^*J*_H–H_ = 7.8 Hz, 4H; Dipp *meta*-C*H*), 6.31 (s, 2H; CNC*H*), 2.71 (hept, ^3^*J*_H–H_ = 6.8 Hz, 4H; C*H*(CH_3_)_2_), 1.47 (d, ^3^*J*_H–H_ = 6.9 Hz, 12H; CH(C*H*_3_)_2_), 1.03 (d, ^3^*J*_H–H_ = 6.9 Hz, 12H; CH(C*H*_3_)_2_), 0.36 (s, 18H; SiC*H*_3_), 0.19 (s, 18H; SiC*H*_3_), −0.08 (d, ^3^*J*_P–H_ = 3.9 Hz, 2H; SnC*H*).

**^13^C{^1^H} NMR (126 MHz, C_6_D_6_):** δ (ppm) 201.64 (*C*[N(Dipp)CH)]_2_), 183.38 (d, ^1^*J*_P–C_ = 94.8 Hz; P*C*O), 145.40 (Dipp *ortho*-Ar*C*), 135.24 (Dipp *ipso*-Ar*C*), 130.78 (Dipp *meta*-Ar*C*), 124.74 (Dipp *para*-Ar*C*), 123.39 (NC*H*), 29.04 (Dipp *C*H(CH_3_)_2_), 24.44 (d, ^TS^*J*­_P–C_ = 4.2 Hz; CH(*C*H_3_)_2_), 10.35 (d, ^2^*J*­_P–C_ = 8.4 Hz; Sn*C*H), 4.88 (d, ^4^*J*­_P–C_ = 4.9 Hz; Si*C*H­_3_), 4.58 (Si*C*H_3_).

**^31^P{^1^H} NMR (162 MHz, C_6_D_6_):** δ (ppm) −358.6 (s, *P*CO).

**^119^Sn{^1^H} NMR (186 MHz, C_6_D_6_):** δ (ppm) 261.2 (d, ^1^*J*_Sn–P_ = 606 Hz).

**IR (solid, cm^−1^):** *ṽ* 1896 (PCO).

**Figure S11.** ^1^H NMR (400 MHz, 293 K) of **3** in C_6_D_6_.

**Figure S12.** ^13^C{^1^H} NMR spectrum (127 MHz, 293 K) of **3** in C_6_D_6_.

**Figure S13.** ^31^P{^1^H} NMR spectrum (162 MHz, 293 K) of **3** in C_6_D_6_.

**Figure S14.** ^119^Sn{^1^H} NMR spectrum (186 MHz, 293 K) of **3** in C_6_D_6_.

**Figure S15.** ATR-FTIR spectrum of **3**.

## 1.1.4. Synthesis of (^Dipp^NacNac)Zn(PCO) (4)

(^Dipp^NacNac)ZnCl·LiCl(Et_2_O)_2_ (200 mg, 0.315 mmol) and [Na(diox)_0.32_][PCO] (42 mg, 0.381 mmol) were dissolved in THF (20 mL). The solution was stirred for 1 h before the volatiles were removed *in vacuo*. The residue was extracted with toluene (30 mL) and filtered. The solvent was removed *in vacuo* followed by washing with hexane (3 mL) to give a tan powder (123.9 mg, 0.229 mmol, 73% yield). Crystals suitable for single-crystal X-ray diffraction were grown by slow evaporation of a benzene solution at room temperature. Anal. calculated for C_30_H_41_N_2_OPZn: C, 66.48; H, 7.62; N, 5.17. Found: C, 66.54; H, 7.57; N, 4.99.

**^1^H NMR (400 MHz, C_6_D_6_):** δ(ppm) 7.15 – 7.08 (m, 6H; Dipp ArC*H*), 4.98 (s, 1H; γ-C*H*), 3.12 (hept, ^3^*J*_H–H_ = 7.0 Hz, 4H; C*H*(CH_3_)_2_), 1.66 (s, 6H; NCC*H*_3_), 1.36 (d, ^3^*J*_H–H_ = 6.9 Hz, 12H; Dipp CH(C*H*_3_)_2_), 1.13 (d, ^3^*J*_H–H_ = 6.9 Hz, 12H; Dipp CH(C*H*_3_)_2_).

**^13^C{^1^H} NMR (151 MHz, C_6_D_6_):** δ(ppm) 179.27 (d, ^1^*J*_P–C_ = 101 Hz; P*C*O), 169.19 (*C*=N), 143.13 (Ar*C*), 141.73 (Ar*C*), 127.00 (Ar*C*), 124.31 (Ar*C*), 96.07 (γ-*C*H), 28.73 (Dipp *C*H(CH_3_)_2_), 24.45 (d, *J* = 2.2 Hz; Dipp CH(*C*H_3_)_2_), 23.68 (Dipp CH(*C*H_3_)_2_), 23.46 (NC*C*H_3_).

**^31^P{^1^H} NMR (162 MHz, C_6_D_6_):** δ(ppm) −403.1 ppm (s; *P*CO).

**IR (solid, cm^−1^):** *ṽ* 1907 (PCO).

**Figure S16.** ^1^H NMR spectrum (400 MHz, 293 K) of **4** in C_6_D_6_.

**Figure S17.** ^13^C{^1^H} NMR spectrum (151 MHz, 293 K) of **4** in C_6_D_6_.

**Figure S18.** ^31^P{^1^H} NMR spectrum (162 MHz, 293 K) of **4** in C_6_D_6_.

**Figure S19.** ATR-FTIR spectrum of **4**.

## 1.2.1. Alternative synthesis of Sn[CH(SiMe_3_)_2_]_2_

A Schlenk tube was charged with SnCl_2_ (323 mg, 1.704 mmol) and Mg(Cl)(OEt_2_)CH(SiMe_3_)_2_ (1.0 g, 3.410 mmol, 2 eq.). Addition of Et_2_O (20 mL) caused the solution to change colour to yellow, and then red. The solution was heated to reflux overnight, causing it to become red/purple. The volatiles were removed *in vacuo* and the residue extracted with hexane (100 mL). Following filtration, a red/purple solution was obtained. Concentration of this solution by approximately 50% followed by storage at −20 °C overnight yielded red crystals (308 mg, 0.7040 mmol). A second crop of crystals was obtained by concentrating the filtrate (101 mg, 0.2309 mmol). Combined yield: 409 mg, 0.9348 mmol, 54.9%.

**^1^H NMR (C_6_D_6_):** δ(ppm) 1.77 (2H, SnC*H*), 0.24 (36H, SiC*H*_3_).

The NMR data are in agreement with those previously reported.^[65]^

**Figure S20.** ^1^H NMR (400 MHz, 293 K) spectrum of Sn[CH(SiMe_3_)_2_]_2_ in C_6_D_6_. Inset: expansion of SiC*H*_3_ resonance.

## 1.2.2. Decomposition of 1 above −35 °C

**Method A**: A recrystallised sample of **1** (15.6 mg, 0.015 mmol) was dissolved in C_6_D_6_ (0.5 mL) and left to stand at room temperature overnight. The NMR spectra were recorded without further purification, showing the formation of free ^Dipp^PDI ligand and CH_2_(SiMe_3_)_2_, along with several unidentified species.

**Method B**: (^Dipp^PDI)Co(PCO) (10 mg, 0.017 mmol) and Sn[CH(SiMe_3_)_2_]_­2_ (7.3 mg, 0.017 mmol) were combined in C_6_D_6_ (0.5 mL) and heated at 40 °C for 2 hours. The NMR spectra were recorded without further purification, showing a similar product distribution to that given by Method A.

**^31^P{^1^H} NMR (C_6_D_6_):** δ(ppm) −350.6 (s).

Despite repeated attempts, it has not been possible to identify the species responsible for the ^31^P NMR resonance.

**Figure S21.** ^31^P{^1^H} NMR spectrum (162 MHz, 293 K) after a recrystallised sample of **1** was left to stand overnight in C_6_D_6_. Inset: Expansion of resonance, showing ^117^Sn/^119^Sn satellites.

**Figure S22.** ^1^H NMR spectrum (400 MHz, 293 K) after a recrystallised sample of **1** was left to stand overnight in C_6_D_6_. Peaks corresponding to free ^Dipp^PDI are marked ‘A’. Inset: Expansion of SiCH_3_ region.

**Figure S23.** ^31^P{^1^H} NMR spectrum (162 MHz, 293 K) after a sample of **1** prepared *in situ* was heated at 40 °C for 2 hours in C_6_D_6_.

**Figure S24.** ^1^H NMR spectrum (400 MHz, 293 K) after a sample of **1** prepared *in situ* was heated at 40 °C for 2 hours in C_6_D_6_. Peaks corresponding to free ^Dipp^PDI are marked ‘A’.

## 1.2.3. Reaction of 1 with (IDipp)AuCl

(IDipp)AuCl (3 mg, 0.005 mmol) and recrystalised **1** (5 mg, 0.005 mmol) were combined in C_6_D_6_ (0.5 mL). The green solution was sonicated for 2 minutes and monitored by *in*-*situ* NMR spectroscopy. The formation of **2** was observed by ^31^P NMR spectroscopy after one hour. After two hours, the solution became purple and a mixture of **2** and **3** was observed, followed by conversion to **3** over 18 hours. The formation of (^Dipp^PDI)CoCl was also observed by ^1^H NMR spectroscopy.

**Figure S25.** ^31^P{^1^H} NMR spectra (162 MHz, 293 K) for the reaction of **1** with (IDipp)AuCl in C_6_D_6_ (A: **1**; B: **2**; C: **3**).

**Figure S26.** ^1^H NMR spectrum (400 MHz, C_6_D_6_) for the reaction of **1** with (IDipp)AuCl in C_6_D_6_ after 18 hours. The resonances marked ‘A’ correspond to (^Dipp^PDI)CoCl.

## 1.2.4. Reaction of 1 with (^Dipp^NacNac)ZnCl·LiCl(OEt_2_)_2_

(^Dipp^NacNac)ZnCl·LiCl(OEt_2_)_2_ (4.5 mg, 0.006 mmol, in excess) and recrystalised **1** (5 mg, 0.005 mmol) were combined in C_6_D_6_. The green solution was sonicated for 2 minutes and monitored by *in*-*situ* NMR spectroscopy. The gradual formation of **4** was observed after several hours, along with (^Dipp^PDI)CoCl. The decomposition of **1** was also observed due to the slower transmetallation compared to the reaction to form **2**.

**Figure S27.** ^31^P{^1^H} spectra (162 MHz, 293 K) showing gradual formation of **4**. Resonance marked * is an unknown decomposition product of **1** (see Section 1.2.2.).

**Figure S28.** ^1^H NMR spectrum (400 MHz, C_6_D_6_) for the reaction of **1** with excess (^Dipp^NacNac)ZnCl·LiCl(OEt_2_)_2_ in C_6_D_6_ after 18 hours. The resonances marked ‘A’ correspond to (^Dipp^PDI)CoCl.

## 1.2.5. Reaction of (^Dipp^PDI)Co(PCO) with (IDipp)AuCl

(^Dipp^PDI)Co(PCO) (10 mg, 0.017 mmol) was combined with (IDipp)AuCl (10.3 mg, 0.017 mmol) in C_6_D_6_ (0.5 mL). After shaking the solution for 2 minutes, the NMR spectra were recorded without further purification, showing the formation of a mixture of (^Dipp^PDI)CoCl and **2**.

**Figure S29.** ^31^P{^1^HP NMR spectrum (162 MHz, 293 K) for the reaction of (^Dipp^PDI)Co(PCO) and (IDipp)AuCl in C_6_D_6_, showing formation of **2**.

**Figure S30.** ^1^H NMR spectrum (400 MHz, 293 K) for the reaction of (^Dipp^PDI)Co(PCO) and (IDipp)AuCl in C_6_D_6_. The resonances marked ‘A’ correspond to (^Dipp^PDI)CoCl.

## 1.2.6. Reaction of (^Dipp^PDI)Co(PCO) with (^Dipp^NacNac)ZnCl·LiCl(OEt_2_)_2_

(^Dipp^PDI)Co(PCO) (10 mg, 0.017 mmol) was combined with an excess of (^Dipp^NacNacZnCl·LiCl(OEt_2_)_2_ (12.5 mg, 0.018 mmol) in C_6_D_6_ (0.5 mL). After sonicating the solution for 30 minutes, the precipitation of solids was noted, and the NMR spectra were recorded without further purification, showing the formation of a mixture of (^Dipp^PDI)CoCl and **4**.

**Figure S31.** ^31^P{^1^H} NMR spectrum (162 MHz, 293 K) for the reaction of (^Dipp^PDI)Co(PCO) and (^Dipp^NacNac)ZnCl·LiCl(OEt_2_)_2_ in C_6_D_6_, showing formation of **4**.

**Figure S32.** ^1^H NMR spectrum (400 MHz, 293 K) for the reaction of (^Dipp^PDI)Co(PCO) and (^Dipp^NacNac)ZnCl·LiCl(OEt_2_)_2_ in C_6_D_6_. The resonances marked ‘A’ correspond to (^Dipp^PDI)CoCl.

# 2. Single crystal X-ray diffraction data

Single-crystal X-ray diffraction data were collected using an Oxford Diffraction Supernova dual-source diffractometer equipped with a 135 mm Atlas CCD area detector. Crystals were selected under Paratone-N oil, mounted on micromount loops and quench-cooled using an Oxford Cryosystems open flow N_2_ cooling device. Data were collected using mirror monochromated Cu Kα (λ = 1.54184 Å) or Mo Kα (λ = 0.71073 Å) radiation and processed using the CrysAlisPro package, including unit cell parameter refinement and inter-frame scaling (which was carried out using SCALE3 ABSPACK within CrysAlisPro).^[66]^ Structures were subsequently solved using direct methods and refined on *F*^2^ using the SHELXL package.^[67,68]^

**Table S1.** Selected X-ray data collection and refinement parameters for **1**, **2**, **3**, and **4**.

|  | **1** | **2** | **3** | **4** |
| --- | --- | --- | --- | --- |
| Formula | C_48_H_81_CoN_3_OPSi_4_Sn | C_28_H_36_AuN_2_OP | C_42_H_74_AuN_2_OPSi_4_Sn | C_30_H_41_N_2_OPZn |
| CCDC | 2443222 | 2443223 | 2443224 | 2443225 |
| Fw [g mol^–1^] | 1037.10 | 644.52 | 1082.01 | 541.99 |
| Crystal system | monoclinic | monoclinic | monoclinic | monoclinic |
| Space group | *P*2_1_/*n* | *C*2/*c* | *P*2_1_/*n* | *P*2_1_/*n* |
| *a* (Å) | 14.2961(1) | 16.3381(4) | 18.9807(5) | 15.5120(1) |
| *b* (Å) | 18.8133(1) | 9.9437(2) | 12.5573(2) | 9.3276(1) |
| *c* (Å) | 20.3089(1) | 17.2697(3) | 23.1055(7) | 20.8113(1) |
| α (°) | 90 | 90 | 90 | 90 |
| β (°) | 96.683(1) | 90.152(2) | 110.252(3) | 93.730(1) |
| γ (°) | 90 | 90 | 90 | 90 |
| *V* (Å^3^) | 5425.10(6) | 2805.65(10) | 5166.7(2) | 3004.80(4) |
| *Z* | 4 | 4 | 4 | 4 |
| Radiation, λ (Å) | Cu Kα, 1.54184 | Mo Kα, 0.71073 | Cu Kα, 1.54184 | Cu Kα, 1.54184 |
| Temp (K) | 150(2) | 150(2) | 150(2) | 150(2) |
| ρ_calc_ (g cm^–3^) | 1.270 | 1.526 | 1.391 | 1.198 |
| μ (mm^–1^) | 7.443 | 5.322 | 10.508 | 1.803 |
| Reflections collected | 132963 | 22777 | 115354 | 76313 |
| Independent reflections | 11311 | 3773 | 10829 | 6272 |
| Parameters | 554 | 168 | 473 | 348 |
| R(int) | 0.0373 | 0.0304 | 0.1110 | 0.0231 |
| R1/wR2,^[a]^ I ≥ 2σI (%) | 2.38/5.88 | 1.62/3.58 | 5.66/15.26 | 2.58/7.84 |
| R1/wR2,^[a]^ all data (%) | 2.57/6.01 | 1.79/3.63 | 6.33/16.14 | 2.68/7.93 |
| GOF | 1.019 | 1.074 | 1.056 | 1.062 |

R1 = [Σ||F_o_| – |F_c_||]/Σ|F_o_|; wR2 = {[Σw[(F_o_)^2^ – (F_c_)^2^]^2^]/[Σw(F_o_^2^)^2^}^1/2^; w = [σ^2^(F_o_)^2^ + (AP)^2^ + BP]^–1^, where P = [(F_o_)^2^ + 2(F_c_)^2^]/3 and the A and B values are 0.0282 and 3.96 for **1**·0.5hex, 0.0162 and 1.30 for **2**·0.5C_6_H_6_, 0.0920 and 24.99 for **3**, and 0.0450 and 0.90 for **4**.

# 3. Calculation of δ(PDI) parameters

| **Table S2.** δ(PDI) values and N_py_–Co bond lengths for (^Dipp^PDI)Co(L) complexes. | | | |
| --- | --- | --- | --- |
|   $\delta\left( \mathrm{PDI} \right)= \left( \frac{d2+d2^{'}}{2} \right)-\left( \frac{d1+d1^{'}+d3+d3^{'}}{4} \right)$ | |  |  |
| Complex | δ(PDI)* | N_py_–Co, Å | Ref. |
| (^Dipp^PDI)Co(CO)(P=Sn[CH(SiMe_3_)_2_]) (**1**) | 0.079(1) | 1.819(1) | – |
| [(^Dipp^PDI)Co(CN*t*Bu)_2_]^+^ | 0.103(2) | 1.819(2) | 43 |
| (^Dipp^PDI)Co(PCO) | 0.092(2) | 1.810(2) | 33 |

*Uncertainties were determined by error propagation, where:

$$\sigma\left( \delta\left( \mathrm{PDI} \right) \right)=\sqrt{\left( \frac{\sigma(d2)}{2} \right)^{2}+\left( \frac{\sigma(d2')}{2} \right)^{2}-\left( \frac{\sigma\left( d1 \right)}{4} \right)^{2}{-\left( \frac{\sigma\left( d1^{'} \right)}{4} \right)}^{2}{-\left( \frac{\sigma\left( d3 \right)}{4} \right)}^{2}-\left( \frac{\sigma(d3')}{4} \right)^{2}}$$

# 4. References

[60] C. C. Hojilla Atienza, C. Milsmann, E. Lobkovsky, P. J. Chirik, “Synthesis, Electronic Structure, and Ethylene Polymerization Activity of Bis(imino)pyridine Cobalt Alkyl Cations” *Angew. Chem. Int. Ed.* **2011**, *50*, 8143–8147.

[61] F. Nahra, N. V. Tzouras, A. Collado, S. P. Nolan, “Synthesis of N-heterocyclic carbene gold(I) complexes” *Nat. Protoc.* **2021**, 1476–1493.

[62] D. Heift, Z. Benkő, H. Grützmacher, “Coulomb repulsion versus cycloaddition: formation of anionic four-membered rings from sodium phosphaethynolate, Na(OCP)” *Dalton Trans.* **2014**, *43*, 831–840.

[63] J. Prust, H. Hohmeister, A. Stasch, H. W. Roesky, J. Magull, E. Alexopoulos, I. Usón, H.-G. Schmidt, M. Noltemeyer, “Synthesis and Structural Characterization of β-Diketoiminate Complexes Containing Three-Coordinate Zinc and Copper Atoms” *Eur. J. Inorg. Chem.* **2002**, *2002*, 2156–2162.

[64] T. Fjeldberg, A. Haaland, B. E. R. Schilling, M. F. Lappert, A. J. Thorne, “Subvalent Group 4B metal alkyls and amides. Part 8. Germanium and tin carbene analogues MR_2_ [M = Ge or Sn, R = CH(SiMe_3_)_2_]: syntheses and structures in the gas phase (electron diffraction); molecular-orbital calculations for MH_2_ and GeMe_2_” *J. Chem. Soc. Dalton Trans.* **1986**, 1551–1556.

[65] P. Jutzi, B. Hielscher, “Reaction of decamethylstannocene with lithium alkyls” *Organometallics* **1986**, *5*, 2511–2514.

[66] *CrysAlisPro*, Agilent Technologies, Version 1.171.41.117a.

[67] G. M. Sheldrick, “SHELXT – Integrated space-group and crystal-structure determination” *Acta Cryst.* **2015**, *A71*, 3–8.

[68] G. M. Sheldrick, “Crystal structure refinement with SHELXL” *Acta Cryst.* **2015**, *C71*, 3–8.
